# Phosphorylated neurofilament heavy chain is a marker of neurodegeneration in Leber hereditary optic neuropathy (LHON)

**Published:** 2008-12-22

**Authors:** John Guy, Gerry Shaw, Fred N. Ross-Cisneros, Peter Quiros, Solange R. Salomao, Adriana Berezovsky, Valerio Carelli, William J. Feuer, Alfredo A. Sadun

**Affiliations:** 1 Bascom Palmer Eye Institute, McKnight Vision Research Center, Miami, FL; 2 Department of Ophthalmology University of Miami Miller School of Medicine, Miami, FL; 3 Department of Neuroscience, McKnight Brain Institute, University of Florida College of Medicine, Gainesville, FL; 4 Department of Ophthalmology, Doheny Eye Institute and Keck-USC School of Medicine, Los Angeles, CA; 5 Department of Neurosurgery, Doheny Eye Institute and Keck-USC School of Medicine, Los Angeles, CA; 6 Department of Ophthalmology, Federal University of Sao Paulo, UNIFESP, Sao Paulo, Brazil; 7 Dipartimento di Scienze Neurologiche, Università di Bologna, Bologna, Italy

## Abstract

**Purpose:**

To determine the profile of neurodegeneration in Leber hereditary optic neuropathy (LHON).

**Methods:**

We quantitated serum levels of phosphorylated neurofilament heavy chain (pNF-H) in a Brazilian pedigree of 16 affected patients and 59 carriers with LHON, both molecularly characterized as harboring the G to A mutation at nucleotide 11,778 of the mitochondrial genome. The association of subject characteristics to pNF-H levels was studied with multiple regression; pNF-H data were square-root transformed to effect normality of distribution of residuals. Relationships between the square-root of pNF-H and age and sex were investigated within groups with Pearson correlation and the two-sample *t*-test. Linear regression was used to assess the difference between groups and to determine if the relationship of age was different between affected individuals and carriers. Results of plotting pNF-H levels by age suggested a nonlinear, quadratic association so age squared was used in the statistical analysis. ANCOVA was used to assess the influence of age and group on pNF-H levels.

**Results:**

In the carrier group, there was a significant correlation of square-root pNF-H (mean=0.24 ng/ml^2^) with age (r=0.30, p=0.022) and a stronger correlation with quadratic age (r=0.37, p=0.003). With a higher mean pNF-H (0.33 ng/ml^2^) for the affected group, correlations were of similar magnitude, although they were not statistically significant: age (r=0.22, p=0.42), quadratic age (r=0.22, p=0.45). There was no correlation between age and pNF-H levels (mean=0.34 ng/ml^2^) in the off-pedigree group: age (r=0.03, p=0.87), quadratic age (r=0.04, p=0.84). There was no difference between sexes and pNF-H levels in any of the groups (affected, p=0.65; carriers, p=0.19; off-pedigree, p=0.93).

**Conclusions:**

Elevated pNF-H released into the serum of some affected LHON patients may suggest that axonal degeneration occurs at some point after loss of visual function. Increases in pNF-H levels of carriers with increasing age, not seen in off-pedigree controls, may suggest subtle subclinical optic nerve degeneration.

## Introduction

Leber Hereditary Optic Neuropathy (LHON) usually presents as a bilateral loss of central vision that typically progresses over weeks to months without pain, until bilateral central scotomas, dyschromatopsia, and severe visual loss remain [1–3]. The mean age of onset is in the mid-20s, although the range is extremely broad. Initially, the optic disc may be swollen and the peripapillary retinal nerve fiber layer edematous, then the optic disc atrophies. A common feature during the acute phase of LHON is peripapillary microangiopathy, which was first described by Leber in 1871 [4]. Histopathology of end-stage autopsied nerves showed axonal loss [5] that likely limits spontaneous recovery of vision. The pattern visual evoked potential is affected in the early stages of LHON and becomes extinguished at the atrophic stage, indicating the loss of function of most retinal ganglion cells [6,7]. Nevertheless, electroretinograms remain normal, suggesting the maintenance of photoreceptor cells, bipolar cells, and the retinal pigment epithelium [8]. Though LHON is typically monosymptomatic and does not limit life-span, in early onset cases (2–4 years) other organ systems are involved, and are characterized by muscle weakness, general dystonic rigidity, impaired speech and intelligence and short stature [9–11].

LHON is the most common mitochondrial disease [12]. Almost 20 years ago, it became the first disorder for which a point mutation in mitochondrial DNA (mtDNA) was linked to a maternally inherited human disease [13]. Initial molecular characterization of LHON revealed a G to A transition at nucleotide 11,778 in mtDNA in the gene specifying the NADH dehydrogenase subunit 4 (*ND4*) of complex I, resulting in an arginine to histidine substitution at amino acid 340 [13]. Since then, approximately 45 other pathogenic point mutations in human polypeptide-coding mtDNA genes have been linked to LHON [14]. Still, only three primary mtDNA mutations (G3460A, G11778A, and T14484C) account for 95% of LHON cases, with the G11778A mutation being the most common, accounting for 50% of LHON cases [15,16]. Patients with the G11778A mutation in mtDNA have the poorest visual prognosis.

Currently, there is no effective therapy for LHON [17] or for any other disease caused by mutated mtDNA [18]. Experimental approaches, such as recoding mitochondrial genes in the nuclear genetic code and directing them for import into mitochondria with a targeting sequence, have shown promise in rescuing cultured LHON cells [18–21]. One of the myriad potential problems in applying such promising experimental treatments to patients with LHON is that it is unclear when the degeneration of ganglion cells of the retina and axons of the optic nerve actually begins and when it becomes irreversible. Optical coherence tomography studies have detected thinning of the inferior temporal nerve fiber layer of the retina in asymptomatic carriers with the LHON mutation [22,23]. This finding suggests that loss of axons may occur before the visual loss. If so, then intervention may be needed before optic disc edema sets in, which is seen in LHON patients at the time of visual loss. A mouse model of LHON has shown that axonal and retinal ganglion cell (RGC) loss is not apparent when the optic nerve head is swollen, but that this axonal and RGC loss is advanced 6 months later [24]. Since heavily phosphorylated axonal form of the neurofilament heavy chain (pNF-H) has been shown to be a prospective marker of neurodegeneration [25], we evaluated serum pNF-H levels in a large Brazilian pedigree [26,27] to determine the profile of axonal and RGC loss in LHON patients and asymptomatic carriers of mutated G11778A mtDNA.

## Methods

### Clinical evaluation and molecular characterization

The families (all from one pedigree) were all recruited from a rural valley between the cities of Colatina and Santa Teresa, Brazil. All signed consents and the study was approved of by both the IRBs of Fed. Univ. of Sao Paulo, Brazil and USC, LA, CA. There was a separate consent for blood drawing that was then sent separately and later from Brazil to Los Angeles under the auspices of Brazilian authorities. Descendants through seven generations of an Italian female ancestor born in 1861 were examined by a team of international and Brazilian neuroophthalmologists and research scientists. Clinical examinations included a visual acuity test measured with the EDTRS retro-illuminated “tumbling E” chart and ophthalmoscopy of the optic nerve head and retina. Abnormalities of the optic disc included optic atrophy, microangiopathy or swelling of the optic nerve head. The diagnosis of LHON was made clinically following complaints of visual loss in 16 patients, and the presence of G11778A mtDNA confirmed molecularly from DNA extracted from white blood cells obtained from peripheral blood. Also examined were 59 carriers with the responsible mtDNA mutation, who did not complain of loss of vision. Controls consisted of the spouses of maternally related individuals having neither the mtDNA mutation nor any significant visual problems and male descendents of affected or carrier men.

### pNF-H assay

Serum of G11778A LHON carriers and patients with LHON from a peripheral blood draw in 2005 were coded to mask patient identity. Prior to analysis samples were thawed out and then centrifuged at 13,000x g in an Eppendorf centrifuge to pellet out particulate material. The pNF-H assay was performed essentially as described by Shaw and coworkers [28]. Recently, the laboratories of Drs. Shaw and Petzold have independently described pNF-H capture ELISAs with excellent correlation between the two methods [29]. In brief, the assay was conducted using wells of microtiter plates coated overnight with 100 μl of affinity purified chicken pNF-H capture antibody (EnCor Biotechnology Inc., Gainesville, FL), diluted about 1:40 in 10 ml 0.05 M carbonate buffer, pH 9.5 to give a final concentration of 1 μg chicken IgG per ml. The antibody and carbonate mix was decanted and the plates blocked with 150 μl of 5% nonfat milk in Tris Buffered Saline (TBS) for 1 h. Each plate was washed with 2% nonfat milk in TBS (10 mM Tris, 150 mM NaCl, pH=7.5) and 0.1% Tween-20 (pH 7.5). For storage at 4 °C in a sealed damp box, plates were filled with 50 μl TBS and 0.1% Tween-20 with 1 mM of sodium azide added as preservative. After washing, a total of 50 μl standard or 10 μl serum sample plus 40 μl 2% nonfat milk in TBS and 0.1% Tween-20 were added in duplicate to the plate. The plates were incubated on a shaker at room temperature (RT) for 1 h. After washing, 100 μl affinity purified rabbit anti-pNF-H antibody, at a final concentration of 1 μg/ml in 2% nonfat milk in TBS plus 0.1% Tween-20, was added to each well and the plate incubated for 1 h at RT. The microtiter plate was washed and 100 μl alkaline phosphatase–labeled goat anti-rabbit antibody (Sigma, St. Louis, MO), diluted 1:1000 in TBS, was added and incubated for 1 h at RT. After a final wash, 100 μl pNP phosphatase substrate in 0.1 M glycine, 1 mM Mg^2+^, 1 mM Zn^2+^ at pH 10.4 was added per well. The plate was incubated with shaking at RT. The assay showed low background, and although usable data were obtained within 15 min, the reactions were allowed to proceed for 2 h or more. The absorbance was read at a wavelength of 405 nm on a Tecan SpectraFluor ELISA plate reader (Tecan Group Limited, Durham NC), generally 1 h after addition of chromogen. The reaction was stopped by adding 50 μl 2M NaOH per well.

### Data analysis

Data analysis was performed using SPSS statistical software (Chicago, IL). The association of subject characteristics to pNF-H levels was studied with multiple regression. The pNF-H data were square-root transformed to effect normality of distribution of residuals. Relationships between the square-root of pNF-H and age and sex were investigated within groups with Pearson correlation and the two-sample *t*-test. Linear regression was used to assess the difference between groups and to determine whether the relationship of age was different between affected individuals and carriers. Including both the carrier and affected individuals in the same analysis raised the question of what age was appropriate. We used the age at time of testing for the carriers and the age of onset for the affected individuals. Results of plotting pNF-H levels by age suggested a nonlinear, quadratic association, so age squared was used in the statistical analysis. ANCOVA was used to assess the influence of age and group on pNF-H levels.

## Results

### Clinical profile

The clinical profile of LHON patients is shown in Table 1. Their average age was 42 years with a range from 17 to 68 years. The interval between onset of visual loss and examination ranged from 1 to 33 years, with an average of 18 years. There was a 7:1 male predominance with 14 men and two women. The women were the two oldest LHON patients studied. Visual acuity was poor, ranging from counting fingers to hand movements. Ophthalmoscopy revealed optic atrophy in all patients.

**Table 1:** Clinical profile of LHON patients

**Sex**	**Age (examination)**	**Age (onset)**	**VA OD**	**VA OS**	**Disc OD**	**Disc OS**
M	38	*	CF	CF	pale	pale
M	48	48	CF	CF	pale	pale
M	43	21	CF	CF	pale	pale
M	18	14	CF	CF	pale	pale
M	24	18	CF	CF	pale	pale
M	27	*				
F	68	35	CF	CF	pale	pale
F	62	31	HM	HM	pale	pale
M	50	25	CF	CF	pale	pale
M	41	32	CF	CF	pale	pale
M	46	35	CF	CF	pale	pale
M	17	16	CF	CF	pale	pale
M	35	13	CF	CF	pale	pale
M	52	30	HM	HM	pale	pale
M	59	40	CF	CF	pale	pale
M	48	41	CF	CF	pale	pale

The asterisk indicates missing data. This table describes the summary of sex, age at the time of examination, age of onset of visual loss, visual acuity, and optic disc appearance at the time of examination of LHON patients.

The clinical profile of carriers with mutated mtDNA is shown in Appendix 1. Their average age was 36 years, with a range from 8 to 65 years. There was a 2:1 female predominance with 40 women and 19 men. While visual acuity ranged from 20/20 to counting fingers, most carriers had good vision with a mean visual acuity of 0.109 for the right eyes and 0.107 for the left eyes (ETDRS logMAR charts). We found 23 patients had abnormal ophthalmoscopy with either swelling of the nerve fiber layer, hyperemia, microangiopathy, or pallor of the optic nerve head. There were 17 men and 22 women in the off-pedigree control group. Their average age was 39 years. They had no ocular diseases.

### pNF-H titers

**Figure 1:**
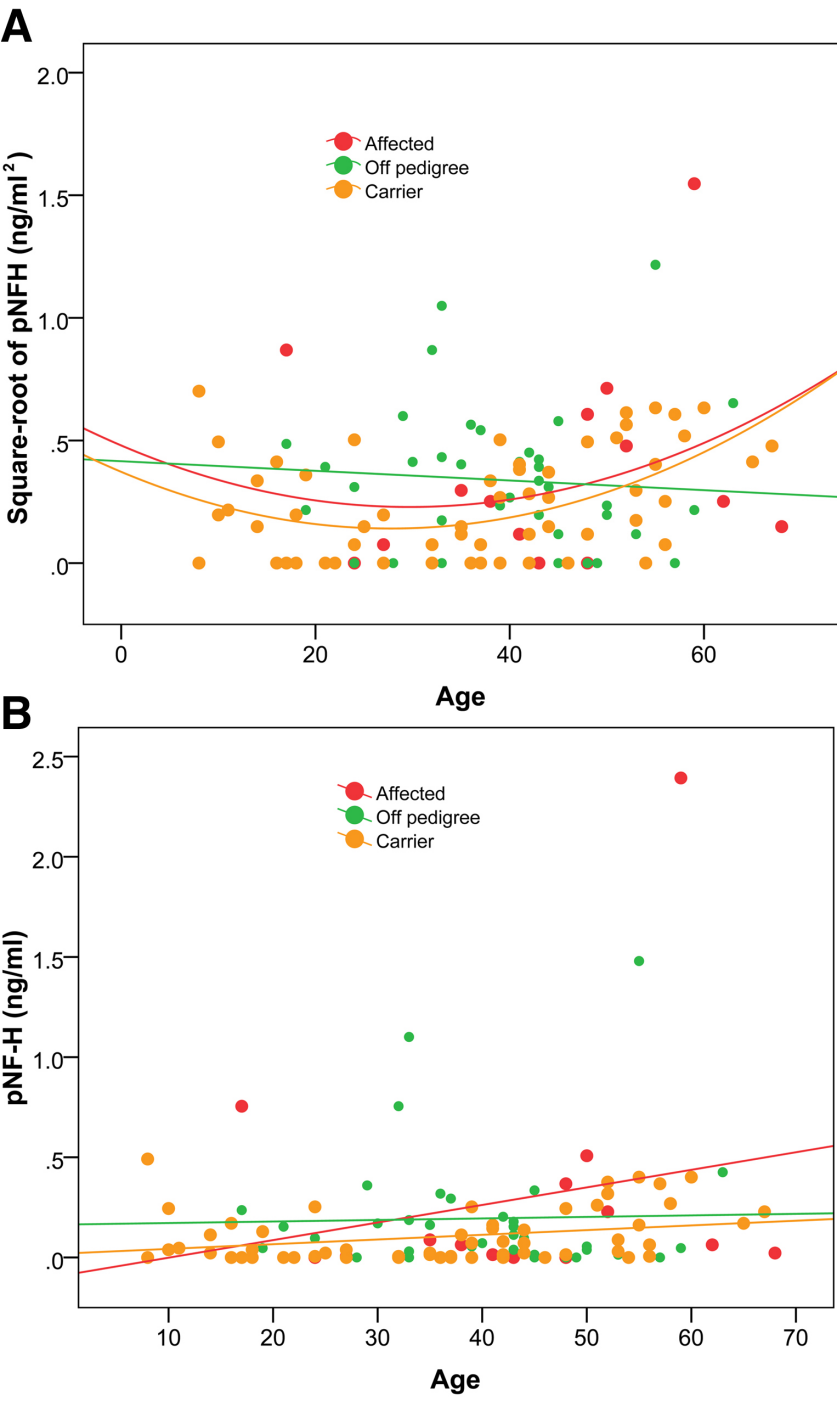
pNF-H levels versus age. Plots of the square root of pNF-H (ng/ml^2^; **A**) and pNF-H (ng/ml; **B**) show that pNF-H levels increase with advancing age of carriers and affected patients but not in the off-pedigree control group.

Figure 1 shows plots of square-root pNF-H (Figure 1A) and pNF-H (Figure 1B) in affected individuals and carriers by decade of age. In the carrier group there was a significant correlation of square-root pNF-H with age (r=0.30, p=0.022) and a stronger correlation with quadratic age (r=0.37, p=0.003). Correlations were of similar magnitude but not statistically significant in the smaller affected group: age (r=0.22, p=0.42), quadratic age (r=0.22, p=0.45). There was no correlation between age and pNF-H levels in the off-pedigree group: age (r=0.03, p=0.87), quadratic age (r=0.04, p=0.84). There was no difference between sexes in pNF-H levels in any of the groups (affected, p=0.65; carriers, p=0.19; off-pedigree, p=0.93)

Quadratic age was significantly correlated with square-root of pNF-H (p=0.012), and the affected group was elevated above the carrier group (p=0.044). The association of quadratic age in carriers and affected groups was not significantly different (p=0.80, test of group by age interaction). This relationship is displayed in Figure 1. Off-pedigree individuals displayed no evidence of this relationship, and they had pNF-H measurements that were comparable to those of affected individuals.

## Discussion

We have shown here that phosphorylated neurofilament heavy chain appears to be a marker of neurodegeneration in affected LHON patients relative to asymptomatic carriers with mutated mtDNA. Neurofilaments are major components of the axonal cytoskeleton that consist of 3 major subunits: a light (NF-L), medium (NF-M), and heavy (NF-H) chain. They are released into the bloodstream with axonal disruption, and the heavily phosphorylated axonal form of NF-H, pNF-H, has a collection of unusual properties that render it resistant to proteases and relatively easy to detect with ELISA and other antibody-based assays. That the heavy chain breakdown product was detectable decades after visual loss may suggest that the optic nerve neurodegeneration of LHON may be a lifelong process. This is supported by histopathologic and ultrastructural studies of autopsied LHON patients that show axons of the optic nerve in various stages of degeneration, in addition to the virtually complete axonal loss of the papillomacular bundle [5,30]. Unfortunately, here we were unable to obtain blood samples from patients when they initially lost vision. Still the single sample obtained from a patient within the first year of visual loss revealed undetectable levels of pNF-H. This may suggest that irreversible anatomic axonal injury may not present at the time of acute visual loss in some LHON patients.

Parallels of our patients with the LHON animal model reveal the optic disc edema characteristic of acute visual loss is not immediately associated with any significant loss of axons or their neuronal cell bodies [24]. These experimental findings together with the lack of pNF-H elevation in the single LHON patient examined during the first year of visual loss suggest that axonal loss in patients with acute LHON and disc edema has not yet occurred to any significant degree. Moreover, increased blood levels of pNF-H in some LHON patients suggest not all axons are lost but a number are still undergoing degeneration even many years after visual loss. These findings may suggest the potential of a longer window of opportunity for therapeutic intervention than is currently believed. Still, it must be pointed out that a purported neuroprotectant, brimonidine, was not demonstrated to be effective in preventing loss of vision even when administered topically before significant visual loss during the period of disc edema [17].

The increasing pNF-H blood levels with age of LHON carriers and reactive oxygen species found in the optic nerve of the LHON mouse [24] suggest that accumulating oxidative injury contributes to the neurodegeneration associated with mutated mtDNA. These findings point to two potential avenues of intervention in the disease process. First, antioxidant gene therapy may be useful to rescue patients with LHON from the axonal and neuronal injury responsible for persistent loss of vision [31]. Though it had no counterpart in human disease, rescue of an animal model of complex I deficiency induced by the NDUFA1 ribozyme with a gene that neutralizes reactive oxygen species (mitochondrial superoxide dismutase) proved that in that model system, suppression of reactive oxygen species inhibited death of retinal ganglion cells, a phenomenon that we showed in the LHON animal model is also involved in the pathogenesis of neurodegeneration caused by the mutant human *ND4* complex I subunit gene [24]. Second, complementation of the defective *ND4* subunit gene with the normal *ND4* gene holds promise as an alternate way to treat LHON. We and others have used allotopic complementation with wild-type human ND4 to rescue cultured LHON cells [19,21]. That allotopic expression can rescue complex I deficiency in vivo was proven in a murine model of Parkinson disease. Rather than complementing the defective 8 kDa complex I subunit [32] with a human gene, the investigators used the AAV vector to deliver the single-subunit NADH dehydrogenase (NDI1) of yeast (Saccharomyces cerevisiae) [33]. Despite the marked mismatch in the amino acid sequence and size of the yeast relative to the murine complex I, a 50% rescue of complex I activity was seen in their mice. They have also used the yeast NDI1 to rescue complex I deficiency in a mutant *ND4* cell line [34]. While other experimental approaches such as importing genes from other species, changing the ratio of heteroplasmy with specific restriction endonucleases, selecting for respiratory function or regeneration (in muscle), none of these techniques are directly applicable to the treatment of LHON caused by 100% mutated mtDNA [20,35]. Whether allotopic complementation or antioxidant gene therapy is effective in treating or preventing LHON remains to be determined.

In LHON it is unclear when the degeneration of ganglion cells of the retina and axons of the optic nerve actually begins and when it becomes irreversible. In other diseases, optical coherence tomography has revealed loss of axons in optic neuritis and multiple sclerosis (MS) patients, but could not detect neurodegeneration until after axons were gone, 3–6 months after acute visual loss [36]. In contrast, serum titers of pNF-H have been shown to be a prospective marker of neurodegeneration in optic neuritis and multiple sclerosis patients [37–39]. In those patients, elevated pNF-H titers carried a poor visual prognosis as was also the case with our LHON patients, who had been followed clinically for 5 years and had exhibited no evidence for visual recovery. Whether asymptomatic carriers with elevated pNF-H titers will progress to full blown LHON is unclear. In MS, cerebrospinal fluid pNF-H levels increased in those with primary or secondary progressive disease, but in those with relapsing remitting disease pNF-H titers were elevated acutely and decreased later [40]. These findings suggest that the patterns of neurodegeneration differ, even in a single disease such as MS. This may also be the case for LHON. Currently, the prognosis for asymptomatic family members with mutated mtDNA is unclear, despite many studies that have looked for additional genetic markers and environmental factors [27,41–43]. It will be intriguing to follow asymptomatic carriers using the pNF-H assay to determine whether the increases with advancing age will progress to frank disease or whether the progression of neurodegeneration in carriers progresses too slowly to advance beyond the threshold of clinical visual loss in their lifetime.

## Appendix 1. Clinical profile of carriers

To access the data, click or select the words “Appendix 1.” This will initiate the download of a compressed (pdf) archive that contains the file. The asterisk indicates missing data. This appendix describes the summary of sex, age at the time of examination, age of onset of visual loss, visual acuity, and optic disc appearance at the time of examination of asymptomatic carriers with G11778A mutated mtDNA.
